# Genome-wide sequence variations between wild and cultivated tomato species revisited by whole genome sequence mapping

**DOI:** 10.1186/s12864-017-3822-3

**Published:** 2017-06-02

**Authors:** Kamlesh Kumar Sahu, Debasis Chattopadhyay

**Affiliations:** National Institute of Plant Genome Research, Aruna Asaf Ali Marg, New Delhi, 110067 India

**Keywords:** *Solanum lycopersicum*, Wild tomato, Genome, SNP, Domestication

## Abstract

**Background:**

Cultivated tomato (*Solanum lycopersicum* L.) is the second most important vegetable crop after potato and a member of thirteen interfertile species of *Solanum* genus. Domestication and continuous selection for desirable traits made cultivated tomato species susceptible to many stresses as compared to the wild species. In this study, we analyzed and compared the genomes of wild and cultivated tomato accessions to identify the genomic regions that encountered changes during domestication.

**Results:**

Analysis was based on SNP and InDel mining of twentynine accessions of twelve wild tomato species and forty accessions of cultivated tomato. Percentage of common SNPs among the accessions within a species corresponded with the reproductive behavior of the species. SNP profiles of the wild tomato species within a phylogenetic subsection varied with their geographical distribution. Interestingly, the ratio of genic SNP to total SNPs increased with phylogenetic distance of the wild tomato species from the domesticated species, suggesting that variations in gene-coding region play a major role in speciation. We retrieved 2439 physical positions in 1594 genes including 32 resistance related genes where all the wild accessions possessed a common wild variant allele different from all the cultivated accessions studied. Tajima’s D analysis predicted a very strong purifying selection associated with domestication in nearly 1% of its genome, half of which is contributed by chromosome 11. This genomic region with a low Tajima’s D value hosts a variety of genes associated with important agronomic trait such as, fruit size, tiller number and wax deposition.

**Conclusion:**

Our analysis revealed a broad-spectrum genetic base in wild tomato species and erosion of that in cultivated tomato due to recurrent selection for agronomically important traits. Identification of the common wild variant alleles and the genomic regions undergoing purifying selection during cultivation would facilitate future breeding program by introgression from wild species.

**Electronic supplementary material:**

The online version of this article (doi:10.1186/s12864-017-3822-3) contains supplementary material, which is available to authorized users.

## Background

Domestication can be regarded as selection of suitable wild accessions according to phenotype and nutrition quality followed by subsequent distribution through human migration. It resulted in recurrent selection of a few loci for better yield, quality and adaptability culminating into purification and diversification (positive selection) of some of the loci, subsequently leading into varietal difference. High throughput DNA sequencing has enabled tracking those selections and facilitated crop improvement [1–5]. Tomato (*Solanum lycopersicum*) belongs to nightshade family and one of the leading vegetable crops. Its domestication has been studied intensely [6–11]. It is a member of 13 interfertile species and having centre of origin in South America [9, 12]. The cultivated tomato is predominantly selfed and highly inbred, and was domesticated from its wild progenitor *S. pimpinellifolium*. The first domesticated cultivar is presumably represented by *S. lycopersicum* var. *cerasiforme* [13]. Tomato was already domesticated during the arrival of the Europeans in South America. The Europeans re-introduced tomato cultivars into Americas about three hundred years ago. Wild tomatoes have large genetic and phenotypic diversity, which is decimated in the cultivated varieties due to domestication bottlenecks [14]. The most studied domestication trait associated with tomato cultivation is its fruit size and shape [15–18]. However, there are several agronomically important traits that have been compromised in the present cultivars. Tomato hosts more than 200 species of pests and pathogens eg. *Cladosporium fulvum* [19], *Xanthomonas performe* [20] and Tomato leaf curl virus [21] which cause significant crop loss. Resistance sources have been identified in the wild relatives, in particular in *S. pimpinellifolium*, *S. peruvianum*, *S. hirsutum* and *S. habrochaitis* [22, 23]. Tomato grows in subtropical region and most of the commercial varieties are sensitive to different environmental stresses, including salinity, drought, excessive moisture etc. There is limited genetic variation for abiotic stress tolerance within the cultivated species. Sources for genetic tolerance to abiotic stresses are available in some wild species such as, *S. pennellii*, *S. chilense* and *S. peruvianum*. As stated above, domestication was mainly focused on increasing yield and size of tomato. Apart from that, various commercial attributions were introgressed in cultivated tomatoes for example, total soluble solid (TSS) and palatability according to market demands. All these efforts have eroded the genetic variability associated with various useful traits in the cultivated tomatoes [24, 25].

After sequencing of the reference tomato genome, several studies regarding comparative transcriptome, and resequencing of wild and cultivated tomatoes have been reported [26–29]. Sequence and expression patterns of the genes suggested positive selection of genes associated with environmental response and stress tolerance and indicated that human manipulation of the genome impacted tomato transcriptome and indirectly favoured nonsynonymous substitution over synonymous substitution [26]. A study involving resequencing of 360 tomato accessions outlined the course of tomato domestication and identified genomic regions of wild species introgressed into cultivated varieties for improvements [28]. Another study explored genetic variations in 84 selected tomato accessions and wild relatives to present sequence diversity in different subsections of tomato and resolved a phylogenetic relationship among different subsections [29]. We have reanalyzed this sequence information and searched for polymorphic sequences between all the wild species together used in this study and the cultivars in order to scan the erosion of genetic base and selection during domestication.

## Results and discussion

### Species-specific polymorphism in wild tomato species

We have used data of 29 accessions of 12 wild tomato species of different subsections and 40 tomato cultivars for the analysis (Table 1) [29]. A stringent criteria of minimum read depth 5, quality score 30 and 100% non-reference allele was applied for calling polymorphism in the wild species in order to filter out errors and to increase confidence in variant calling. We have analyzed the data of one accession of each wild species mapped on the tomato reference genome to verify whether our filter criteria caused any difference in polymorphism pattern as compared to the previous report [29]. The same polymorphism pattern (SNP and insertion/deletion) was observed following our filtering criteria (Additional file 1: Table S1). Expectedly, sequence variations are less in number in the red-fruited species *S. pimpinellifolium*, *S. cheesmaniae* and *S. galapagense* due to their close relatedness with the cultivated species. Extent of variation increased in the green-fruited *S. chmielewskii*, *S. aracanum*, *S. neorickii* and *S. huaylasense* belonging to Aracanum subsection, however, decreased again in the species belonging to Eriopersicon subsection due to less mapping of the reads on reference genome because of distant relationship [29].

**Table 1:** List of cultivated and wild tomato accessions used in this study

SN	Species	Accessions	Compatibility	Sub-Section
1	*S. lycopersicum* cultivar	EA00157, EA00325, EA00371, EA00375, EA00488, EA00892, EA00940, EA00990, EA01019, EA01037, EA01049, EA01088, EA01155, EA01640, EA02054, LA1090, LA2463, LA2706, LA2838A, LA4451, LYC11, LYC1410, LYC1969, LYC3476, LYC3897, PC11029, PI093302, PI158760, PI169588, PI203232, PI303721, PI406760, TR00003, TR00018, TR00019, TR00020, TR00021, TR00022, TR00023, V710029	SC	Lycopersicon
2	*S. pimpinellifolium*	LA1578, LA1584, LYC2798	Self-Compatible (SC)	
3	*S. cheesmaniae*	LA0483, LA1401	SC	
4	*S. galapagense*	LA1044	SC	
5	*S. chmielewskii*	LA2663, LA2695	SC	Arcanum
6	*S. arcanum*	LA2157, LA2172	Typically Self-Incompatible (SI), rarely SC	
7	*S. neorickii*	LA0735, LA2133	SC	
8	*S. huaylasense*	LA1364, LA1365, LA1983	Typically SI	Eriopersicon
9	*S. peruvianum*	LA1278, LA1954	Typically SI	
10	*S. corneliomuelleri*	LA0118	Typically SI	
11	*S. chilense*	CGN15530, CGN15532	SI	
12	*S. habrochaites*	CGN15791, CGN15792, LA1718, LA1777, LA0407, LYC4, PI134418	Typically SI	
13	*S. pennellii*	LA0716, LA1272	Usually SI, some SC	Neolycopersicon

Common polymorphic sequences among all accessions within a wild tomato species were identified. Number of accessions used within a species varied from one to seven and, therefore, variations in one available accession each for *S. galapagense* and *S. corneliomuelleri* were considered. Percentage of SNPs, which were common among all accessions within a wild species (percent common SNP), was calculated (Additional file 1: Table S2). Interestingly, percent common SNPs varied with reproductive behavior of the species. The typically self-incompatible species such as, *S. pennellii*, *S. aracanum*, *S. huaylasense* and *S. habochaites* displayed low percentage of common SNPs, *S. pennellii* being the lowest, suggesting widest genetic variation among the accessions of a wild species. Among the self-incompatible species, *S. peruvianum* and *S. chilense* showed more than 50% common SNP indicating low variation. The two accessions used each for *S. chmielewskii* and *S. neorickii* were highly similar in their genetic background as more than 90% and 80% of the polymorphic sequences, respectively, in these accessions were common. Within the same subsections, based on percentage of common genomic variations, the accessions of self-incompatible *S. aracanum* were highly divergent. This was also reflected in their SNP-based phylogenetic tree [29].

We retrieved the variations in the genomic regions that encode protein-coding genes (genic variations) using the gene coordinates and observed that in the cases of three red-fruited wild species, the genic variations are less than 10% of total genomic variations indicating most of the variations are in non-genic regions (Additional file 1: Table S3). Interestingly, we observed that the ratio of genic SNP to the total SNPs increased with phylogenetic distance of the wild tomato species from the domesticated *S. lycopersicum*, with *S. habrochaites* showing 43% of all variations are in the gene-coding region being the furthest. This observation corroborates its distant position from *S. lycopersicum* in phylogenetic tree [30]. Phylogenetic trees are constructed following different approaches including whole genome SNPs. Sometimes phylogenetic distance between two accessions is perceived from the number of SNPs they display. However, this may lead to errors due to uneven read mapping for different accessions due to distant phylogenetic relationship. In this case, we have observed that the ratios of genic SNPs to the total SNPs could be better correlated with phylogenetic distances of the wild tomato accessions from the reference tomato accession.

**Fig. 1:**
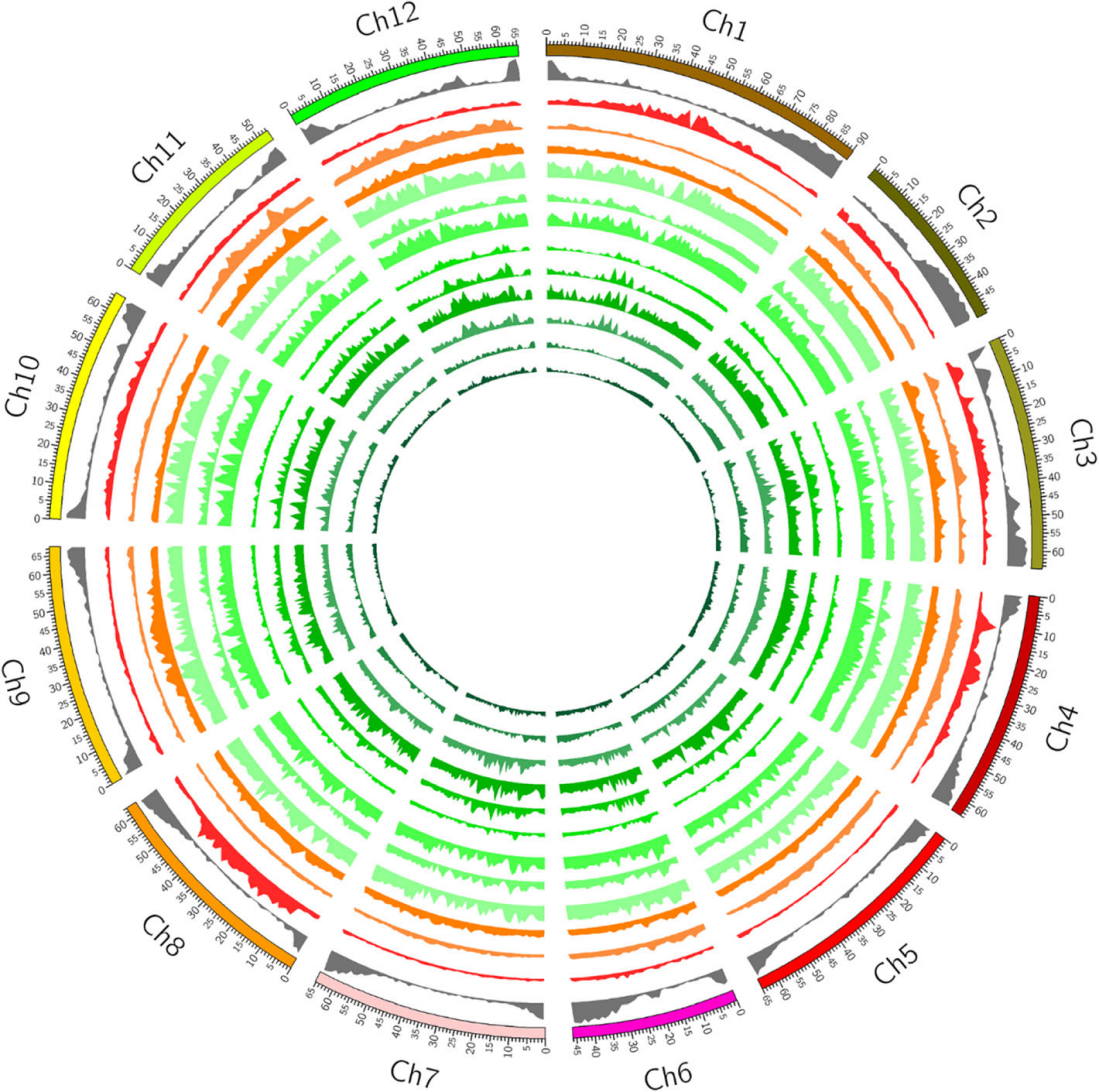
SNPs distribution of 12 wild tomato species on reference tomato chromosomes. The gene and SNPs density have been plotted in 1 Mb sliding window using Circos. The tracks from outside to inside are; chromosomes of tomato; distribution of genes (*gray*) on reference tomato (genes per Mb, max = 180); 12 histogram *circles* of SNPs distribution (max = 14,062) in *S. pimpinellifolium, S. cheesmaniae, S. galapagense, S. chmielewskii, S. arcanum, S. neorickii, S. huaylasense, S. peruvianum, S. corneliomuelleri, S. chilense, S. habrochaites* and *S. pennellii* respectively

**Fig. 2:**
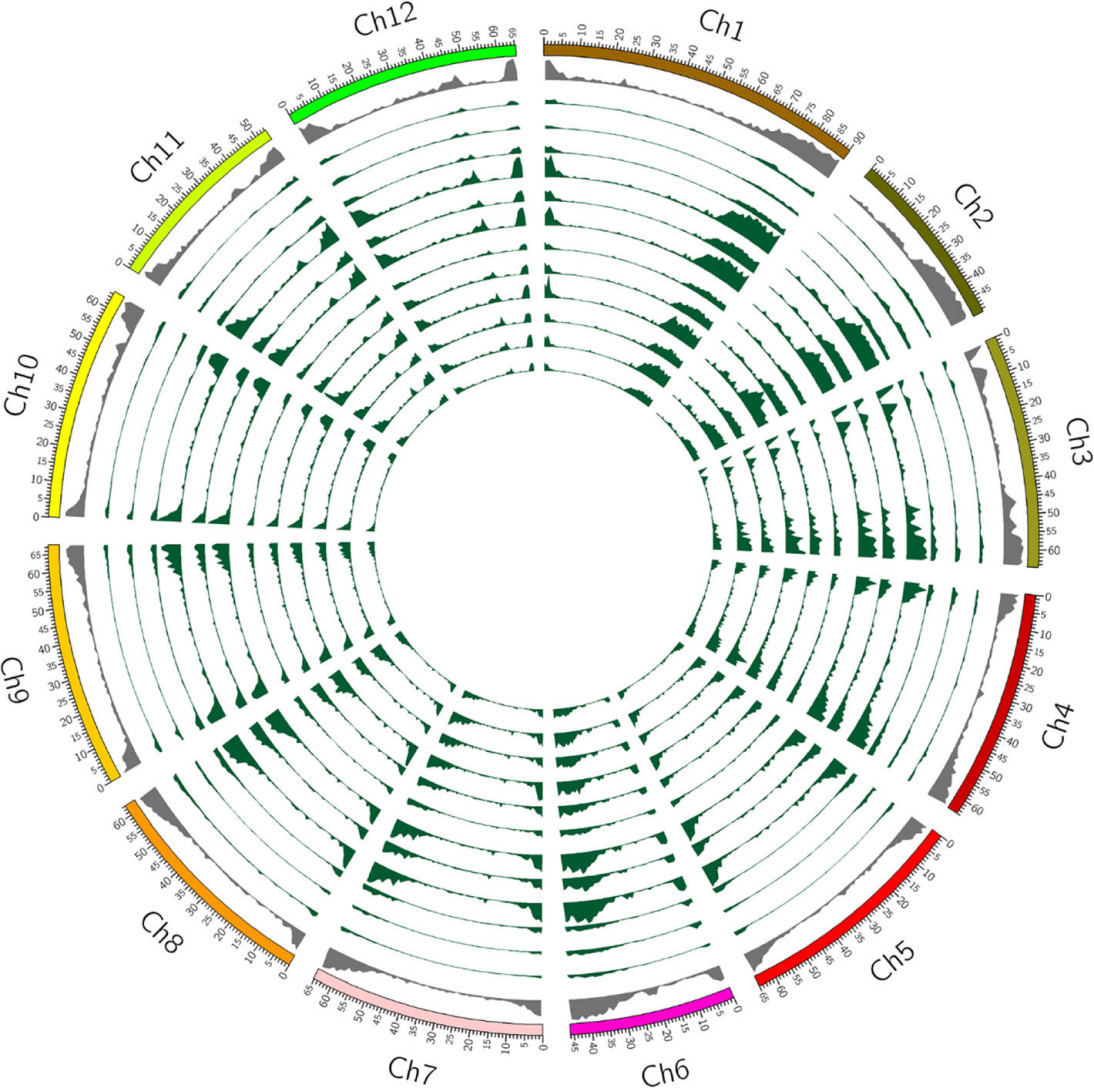
Common genic SNPs of 12 wild tomato species on reference tomato chromosomes. The gene and SNPs density have been plotted in 1 Mb sliding window using Circos. The tracks from outside to inside are; chromosomes of tomato; distribution of genes (*gray*) on reference tomato (genes per Mb, max = 180); 12 *circles* of common genic SNPs distribution (max = 5838) in *S. pimpinellifolium, S. cheesmaniae, S. galapagense, S. chmielewskii, S. arcanum, S. neorickii, S. huaylasense, S. peruvianum, S. corneliomuelleri, S. chilense, S. habrochaites* and *S. pennellii* respectively

Densities of common SNPs over 1 Mb sliding window over the genome were plotted according to their physical positions for each species (Fig. 1). As stated above, data of single accession for *S. galapagense* and *S. corneliomuelleri* were used. A high density of SNP was observed in the heterochromatic regions in chromosomes 1, 3, 4 and 8 in case of *S. pimpinellifolium*, while chromosomes 5 and 7 exhibited two lowest SNP densities (Additional file 2: Table S4). Interestingly, the SNP distribution patterns of *S. cheesmaniae* and *S. galapagense* were very similar, however, entirely different from *S. pimpinellifolium*. These observations is most likely because the *S. cheesmaniae* and *S. galapagense* are the endemic tomato species, or sometimes referred to as two morphotypes rather than two species, of Galapagos Islands and have evolved in isolation from the mainland species, whereas the natural habitat of *S. pimpinellifolium* is the Andean highland [31, 32]. Their different ecological habitats in isolation might have contributed to their genomic diversity. Although, *S. galapagense* and *S. cheesmaniae* shared similar SNP distribution pattern in most of the chromosomes, they differed in case of chromosome 9. The overall common SNP distribution patterns of three species (*S. chmielewskii*, *S. neorickii*, *S. aracanum*) of Aracanum subsections are although similar; the first two (*S. chmielewskii* and *S. neorickii*) bear more similarity than the third one. It is noteworthy to mention that the first two are native of high Andean region whereas *S. aracanum* is a native of coastal and inland Andean region [31]. Additionally, because of self-incompatibility, its genomes appeared to have encountered introgression from different sources. This is supported by the analysis of individual accessions of *S. aracanum* (Additional file 1: Figure S1), which showed the accession LA2172 possessed a very similar SNP distribution pattern to those of *S. chmielewskii* and *S. neorickii*, while LA 2157 possessed different SNP distribution patterns especially in 15–20 Mb region of chromosome 8, 5–7 Mb region of chromosome 9, 40–45 Mb region of chromosome 11 and 25–30 Mb region of chromosome 12. For the species belonging to Eriopersicon subsection, the SNP distribution patterns of *S. peruvianum*, *S. corneliomuelleri* and *S. chilense* showed limited matches especially in chromosomes 1, 6, 7, 9, 10, 12. The common variations in the gene regions were retrieved from the total common variations. *S. chmielewskii* and *S. neorickii* showed high common genic variation because of high similarity of the accessions used, followed by *S. corneliomuelleri* because of one accession used. Interestingly, despite wide sequence divergence among the accessions, *S. habrochaites* showed a high amount of common genic variations. The three species of Lycopersicon subsection expectedly showed low genic variation. In other wild species, common genic SNP distribution along the chromosomes followed the similar patterns and corresponded to gene distribution pattern (Fig. 2). The same distribution pattern was observed for common genic InDels (not shown).

We have identified the common SNPs present in all the accessions of a particular wild tomato species but absent in all other wild and cultivated accessions. To restrict bias for a species, which was represented by only one accession, we have presented data for those wild species, which are represented by at least two accessions. Majority of high-density SNP regions observed in chromosome 1 and 8 in the *S. pimpinellifolium* appears to be specific for these species. Similarly, majority of the species-specific SNPs distributed all over the genome of *S. chmielewskii* appears to be restricted within this species. Eight genes were identified which possessed at least one species-specific nucleotide variation in each of twelve wild tomato species (Additional file 2: Table S5). One of these was CER1 gene (Solyc07g006300.2.1), which is involved in epicuticular wax biosynthesis and pollen fertility [33]. The other genes were GDSL esterase/lipase protein (Solyc12g049550.1.1), involved in floral organ and fertile bud development [34] and sesquiterpene synthase (Solyc06g059930.2.1), responsible for biosynthesis of flower and pollen fragrance [35, 36]. Variability of alleles in these genes related to reproductive development might provide clue for reproductive barrier between different wild tomato species.

### Common genomic erosion during domestication

All the 29 accessions belonging to 12 wild species showed 15,755 common (present in all the wild accessions) sequence variation positions, of which same variant allele was observed in 15,128 positions and different alleles were observed in different accessions in 627 positions. None of these 627 variable allele positions was in genic region. Of 15,128 same common variants, 14,004 were SNPs and 1124 were InDels. Variant call format files of 40 cultivated tomato accessions were analyzed to identify SNP and InDel positions. No quality or read depth filter was applied for the cultivated accessions to maximize the number of sequence variations called. Total 17,125,510 SNPs and 3,610,290 InDels were identified. These sequence variations were in 4,696,093 unique physical positions with respect to the reference genome. Of these, 4,159,616 positions were for SNPs. Chromosome 9 displayed the most diversity with respect to SNP and InDels per Mb followed by chromosome 5, while the lowest diversity was observed in chromosome 10. Chromosomes 1, 2, 3 and 8 also experienced very low sequence diversity per Mb (Additional file 1: Table S6). Most of the SNPs were observed in the heterochromatic or low gene density regions in the chromosomes.

**Fig. 3:**
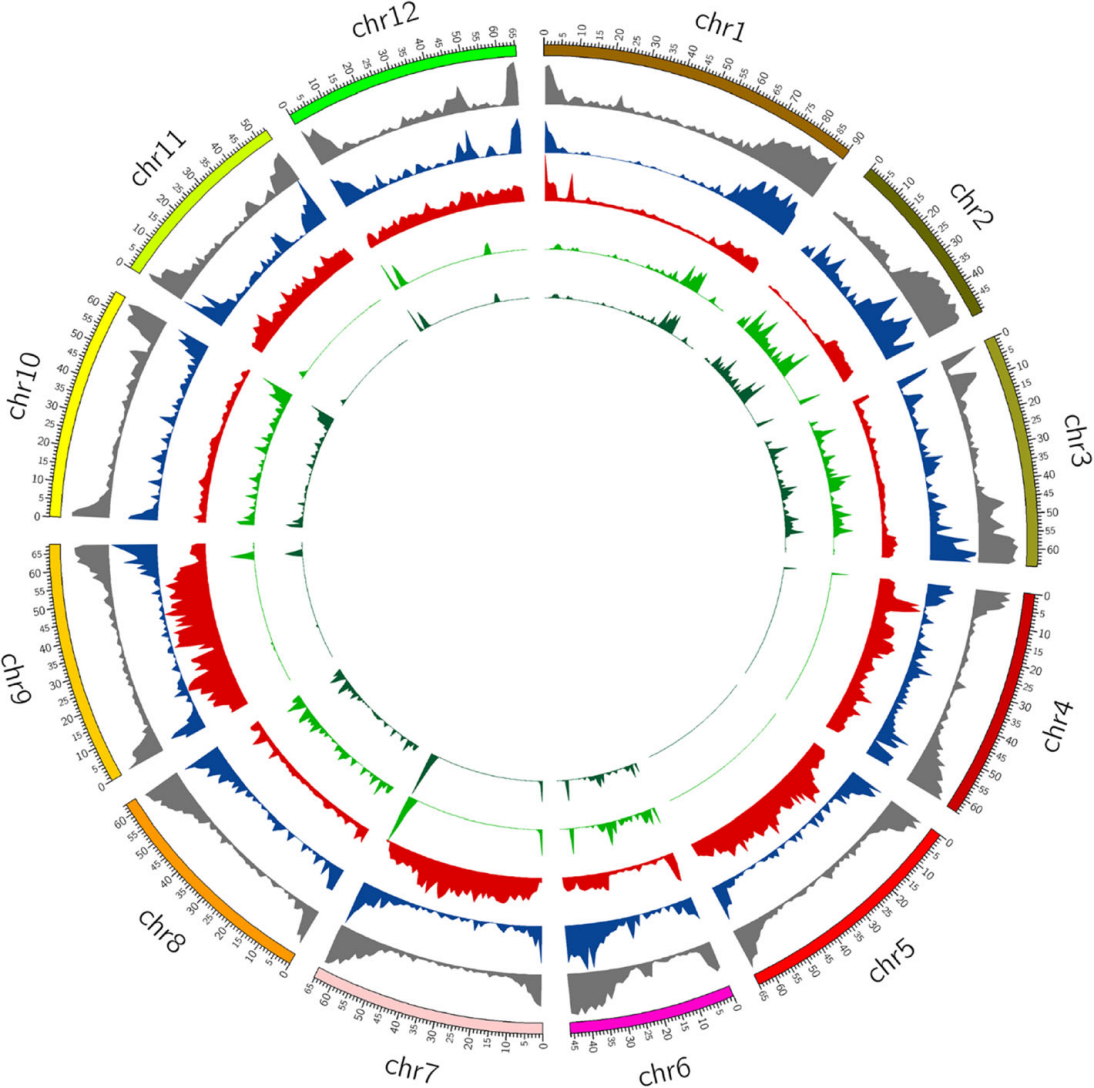
Distribution of common SNPs of wild tomato species and SNPs of cultivated tomato on reference tomato chromosomes. The gene and SNPs density have been plotted in 1 Mb sliding window using Circos. The tracks from outside to inside are; chromosomes of tomato; distribution of genes (*gray*) on reference tomato (genes per Mb, max = 180); common SNPs(*blue*) in 29 accessions of 12 wild tomato species (max = 92); unique SNPs (*red*) present in all cultivated tomato accessions (max = 20,468); common SNPs (*green*) present in 29 accessions of 12 wild tomato but not in present in 40 cultivated tomato accessions (max = 77); common genic SNPs (*dark green*) present in 29 accessions of 12 wild tomato but not present in 40 cultivated tomato accessions (max = 77)

We retrieved 3786 physical positions where all the wild accessions possessed a common variant allele (common wild variant), which was different from all the alleles in all the cultivated accessions. Of those, 3519 were SNPs and 267 were InDels. Most of these common wild variants i.e. 2439 SNPs and 171 InDels resided in only 1594 genes (Fig. 3, Additional file 1: Figure S2). Additionally, eight hundred sixtyeight SNPs were located within 2 kb upstream and downstream regions of the genes. This observation implied that genetic changes associated with domestication and selections have occurred in a few genes. Three-fourth (1192) of these genes were from five chromosomes (Chromosome 1 2, 3, 8 and 10) (Additional file 2: Table S7), which showed very low sequence diversity within the cultivated accessions. None of these genes resided in chromosome 5, which showed very high sequence diversity among the cultivated accessions. More than half (1657) of these common wild variants were in the protein coding regions (CDS) of the genes. These variants were analyzed for their putative effect on the activity of the corresponding genes. Thirty-nine common wild variants showed high putative impact on 39 corresponding genes. Twelve of them were responsible for stop codon loss and 18 SNPs caused stop codon gain, while one SNP was predicted for start codon gain in the cultivated accessions. The other SNPs were predicted for splice variation and two insertions for frame shift (Additional file 2: Table S8).

Domestication is associated with loss of resistance to biotic agents. Therefore, resistance gene homologues (RGHs) including those of receptor like protein kinase (RLK) family were analyzed for the presence of common variants. Thirtytwo genes encoding mostly receptor like kinase proteins and nine NBS-LRR family proteins having common wild variants were obtained (Additional file 2: Table S9). These include genes that code for TMV resistance N-like protein (Solyc01g014840.2.1), Late blight resistance proteins R1A-3 and –R1A-10 (Solyc10g047320.1.1, Solyc01g087200.2.1). Three Mlo genes (Solyc06g010030.2.1, Solyc10g044510.1.1, Solyc08g015870.2.1) also had common variations present in all wild but not present in any cultivated tomato (Additional file 2: Table S7). Mlo protein is a transmembrane protein. This protein family shows an evolutionarily conserved function of susceptibility to powdery mildew. Arabidopsis, tomato, pea and barley plants with mutated Mlo genes showed powdery mildew resistance [37, 38]. Three Mlo genes (Solyc06g010030.2.1, Solyc10g044510.1.1, Solyc08g015870.2.1) had common variations (Additional file 2: Table S7) in all the wild tomato accessions as compared to all the cultivated accessions used in this study.

### Domestication associated selection in tomato genome

**Fig. 4:**
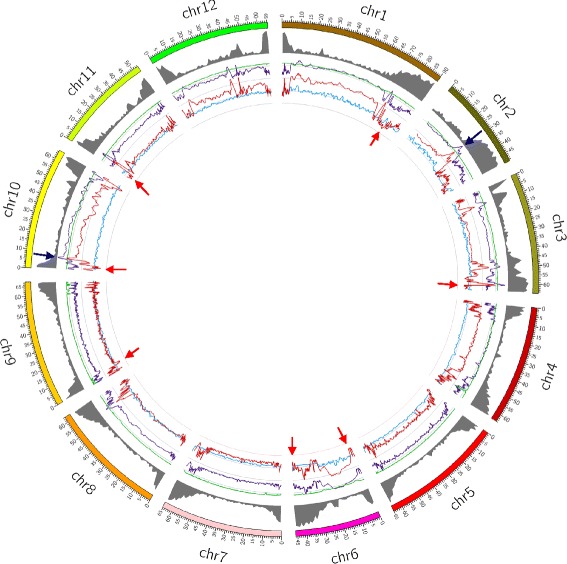
Distribution of Pi, Theta, Fst and Tajima’s D values of 40 cultivated tomato accessions on reference tomato chromosomes. The gene density and Fst have been plotted in 1 Mb sliding window using Circos. Pi, Theta and Tajima’D have been plotted in 500-SNP sliding window with step size 100-SNP. Pi (*Purple*, min = −0.04, max = 0.04); Theta (*Green*, min = −0.04, max = 0.04); Tajima’s D (*Red*, min = −4, max = 4); Fst (*Blue*, min = −0.04, max = 0.04). *Red* and *blue arrows* denote the regions with Tajima’s D value −3 and below and the regions with Tajima’s D value 2 and above, respectively

To understand the genetic diversity in the cultivated tomato accessions nucleotide diversity within a population (Pi) and Tajima’s D in sliding windows were plotted across the genome. High Pi values were associated mostly with the gene-poor repeat-rich regions particularly in chromosome 10. However, a gene-rich region with high value was observed in chromosome 2 (Fig. 4). Consistent with the notion that recent balancing selections for diverse allele contents have occurred in limited genomic regions of tomato, only two genomic regions of tomato, one of 0.5 Mb in length in chromosome 2 and another of 1.3 Mb in length in chromosome 10 with Tajima’s D value equal or more than 2 were obtained. These regions hosted total of seventy genes together. Important genes in this category are three MADS box genes (Solyc10g018070.1.1, Solyc10g018080.1.1, Solyc10g018110.1.1, two of those present tandemly. MADS box proteins are well known for floral development [39]. Two other tandemly repeated genes (Solyc02g062570.2.1, Solyc02g062580.2.1) in encode Dolichyldiphosphatase. One of their orthologs is present in the cultivated soybean (*Glycine max*) but absent from wild soybean (*Glycine soja*). The corresponding protein is known to be involved in acyl lipid metabolism [40]. These regions also host two more tandemly repeated protein pairs, one encoding Pectinesterase inhibitor and the other encoding NHL repeat containing protein. Positive selection in these genes indicates that these regions are recurrently selected for better agronomic traits and resulting in a population contraction within the cultivated tomato.

Cultivated tomato continuously undergoes strong purifying selection to maintain some favoured agronomic trait. Nearly 1% (8.76 Mb) of its genome showed very strong purifying selection with Tajima’s D value less than −3.0. These regions were distributed in seven chromosomes (Additional file 2: Table S10) with more than half (4.578 Mb) of the length contributed by chromosome 11. These regions host a total of 647 genes (Additional file 2: Table S10). Most important of these genes are Auxin Response Factor 9 (ARF9). Larger fruit size is the most important domestication trait in tomato. ARF9 expression in tomato is tightly linked with cell division. Transgenic tomato plant highly expressing ARF9 produced smaller fruit, while the plant with low ARF9 expression produced larger fruit [41]. Two tandemly repeated Gibberellin 2-oxidase 2 (GA2oxs) genes were present in the low Tajima’s D region of chromosome 10. GA2oxs genes act in concert or individually to regulate flowering, tillering and seed germination. Overexpression of C_20_ GAoxs in rice has caused increased root system, higher tiller number and induced semi-dwarfism, which are highly desirable agronomic traits and seems to be under purifying selection in tomato [42]. Six Wax synthase isoform 3 family genes are present in tandem on low Tajima’s D region of chromosome 11. This region also encodes O-acyltransferase and acyl carrier protein. It appears that this genomic region plays a major role in wax biosynthesis in tomato. Wax deposition is a domestication trait as it not only minimize loss of water by transpiration, it also a physical barrier for pathogens. Transcript accumulation of Wax3 genes is enhanced in potato after infection with late blight [43]. A comparison of orthologous genes between *S. lycopersicum* and *S. pennellii* showed an enrichment in acyl carrier proteins in the wild species, which is thought to be important for its desert habitat [44]. Three flowering locus T (phosphatidylethanolamine-binding protein) genes were identified in this region. Of them, two (*SlSP5G2* and *SlSP5G3*) were floral inhibitors and their overexpression delayed flowering [45]. Apart from these, the region with low Tajima’s D value in chromosome 6 hosts two tandemly duplicated genes for Fertility restorer Pentatricopeptide repeat proteins, which are important for restoring cytoplasmic male sterile lines used for hybridization [46, 47]. The potential importance of these genes in agronomic trait improvement might be the reason for purifying selection of this region during domestication.

## Conclusion

We have presented a careful analysis of mapping data of twentynine accessions of twelve wild tomato species and forty accessions of cultivated tomato. Our analysis revealed a diverse polymorphism profile within wild tomato species depicting a rich genetic resource still untapped for tomato improvement. We also observed that within the Aracanum subsection, the accessions of *S. aracanum* species bear highly diverse genomic background as compared to the other two species within the same subsection due to its self-incompatible habit and geographical location. However, the common SNPs of all the accessions within this species and the ratio of common genic SNPs to common genomic SNPs of this species were close to those within same subsection. This observation substantiated that SNPs accumulate mostly in the heterochromatic/gene-poor regions due to change in habitat and the genomic variations within the gene-coding regions are mostly responsible for phenotypic variations leading to speciation. Most of the studies on tomato were focused on fruit shape and size and growth diversification [16–18, 28, 29]. Our analysis was based on overall difference between the wild tomato species and the domesticated accessions at the sequence level. Identification of common wild variants is an important finding. The observation that most of those reside in chromosomes (chromosomes 1, 2, 3, 8, 10) with very low sequence diversity but none in a chromosome (chromosome 5) with very high sequence diversity among the cultivated accessions indicated that chromosomes 1, 2, 3, 8, 10 are important to maintain agronomic traits of the domesticated species, while chromosome 5 can absorb sequence variations including those from the wild species. The genomic regions, which were predicted undergoing positive and purifying selection, and the genes associated with vegetative and reproductive development residing in those locations provide important resource for genetic modification and agronomic improvement.

## Methods

### Getting mapped bam files and vcf files

Previously reported [29] mapping files of the sequence reads of cultivated and wild tomato accessions mapped on reference tomato genome used in this study were downloaded from The European Bioinformatics Institute (EBI), European nucleotide archive accession number PRJEB5235 (http://www.ebi.ac.uk/ena/data/view/ERP004618).

### Common high quality variations

Genomic variations (SNPs and InDels) in 29 accessions of 12 wild tomato species were filtered for high quality, high throughput and homozygosity. High quality variations in each of wild tomato accessions were analysed and compared for common variations within a species and for all wild tomato species. One accession each of *S. corneliomuelleri* and *S. galapagense* was sequenced and thus, only one vcf file of each plants were analyzed. Genic variations were detected using gene coordinates obtained from gff file of reference tomato genome assembly [29]. Common variations within and between the species and species-specific variations were detected using home-made python and bash scripts.

Common high quality SNPs of all wild tomato species were compared with SNP positions of all cultivated tomato accessions without filtering. Common SNPs present in all wild but not present in any cultivated tomato were extracted. Genes possessing these SNPs were detected and its effect were checked through SnpEff [48]. Above procedure were repeated for InDels.

### Tajima’s D test of 40 cultivated tomato

All bam files of 40 domesticated tomato accessions were converted to single bcf file and then vcf file with samtools (var 0.1.19) [49, 50]. This vcf files was converted to hapmap through sniplay [51]. From the hapmap file, diversity were analysed through Tassel (var 5.2.26) [52] with the default parameters (step size – 100, window size – 500) to determine Pi, Theta, Fst and Tajima’s D values.

Stress (biotic and abiotic) resistance related genes (R-genes) were obtained using gff annotation file of reference tomato genome assembly. R-genes with high quality variations present in all wild but not present in any cultivated tomato were detected. Results were represented in circular graph by Circos [53].

## Additional files

Additional file 1: Figure S1. SNPs distribution of wild tomato on reference tomato chromosomes. **Figure S2.** Distribution of common InDels of wild tomato species and InDels of cultivated tomato on reference tomato chromosomes. **Table S1.** High quality variations of one accession each of 12 wild tomato species. **Table S2.** Percentage of common variation within a species. **Table S3.** Common genic and genomic variations in each wild tomato. **Table S6.** Number of SNPs and InDels per 1000 bases chromosome. (PDF 1114 kb)

Additional file 2: Table S4. Common high quality SNPs within each species of wild tomato. **Table S5.** Genes having species specific nucleotide variations in each of twelve wild tomato species. **Table S7.** List of genes with common Variations (SNPs and InDels) present in all wild but not present in any cultivated tomato. **Table S8.** List of genes with high Impact common wild variant alleles. **Table S9.** Variations (present in all wild but absent in all cultivated tomato) and positions of variations in R genes with gene coordinates and gene annotation. **Table S10.** List of genes with less than Tajima’s D value −3. (XLS 482 kb)

## Abbreviations

EBI: European Bioinformatics Institute
InDels: Insertion and deletions
R-genes: Resistance related genes
RGHs: Resistance gene homologues
RLK: Receptor like protein kinase
SC: Self compatible
SI: Self incompatible
SNPs: Single nucleotide polymorphisms
TSS: Total soluble solid
VCF: Variant call format
