# Supplementary material for: Genome-wide sequence variations between wild and cultivated tomato species revisited by whole genome sequence mapping
Source: BMC Genomics. 2017 Jun 2;18:430. doi: 10.1186/s12864-017-3822-3 (PMC5455116; doi:10.1186/s12864-017-3822-3)
Supplement: Supplementary file 1 — SNPs distribution of wild tomato on reference tomato chromosomes. Figure S2. Distribution of common InDels of wild tomato species and InDels of cultivated tomato on reference tomato chromosomes. Table S1. High quality variations of one accession each of 12 wild tomato species. Table S2. Percentage of common variation within a species. Table S3. Common genic and genomic variations in each wild tomato. Table S6. Number of SNPs and InDels per 1000 bases chromosome. (PDF 1114 kb) [file 12864_2017_3822_MOESM1_ESM.pdf]

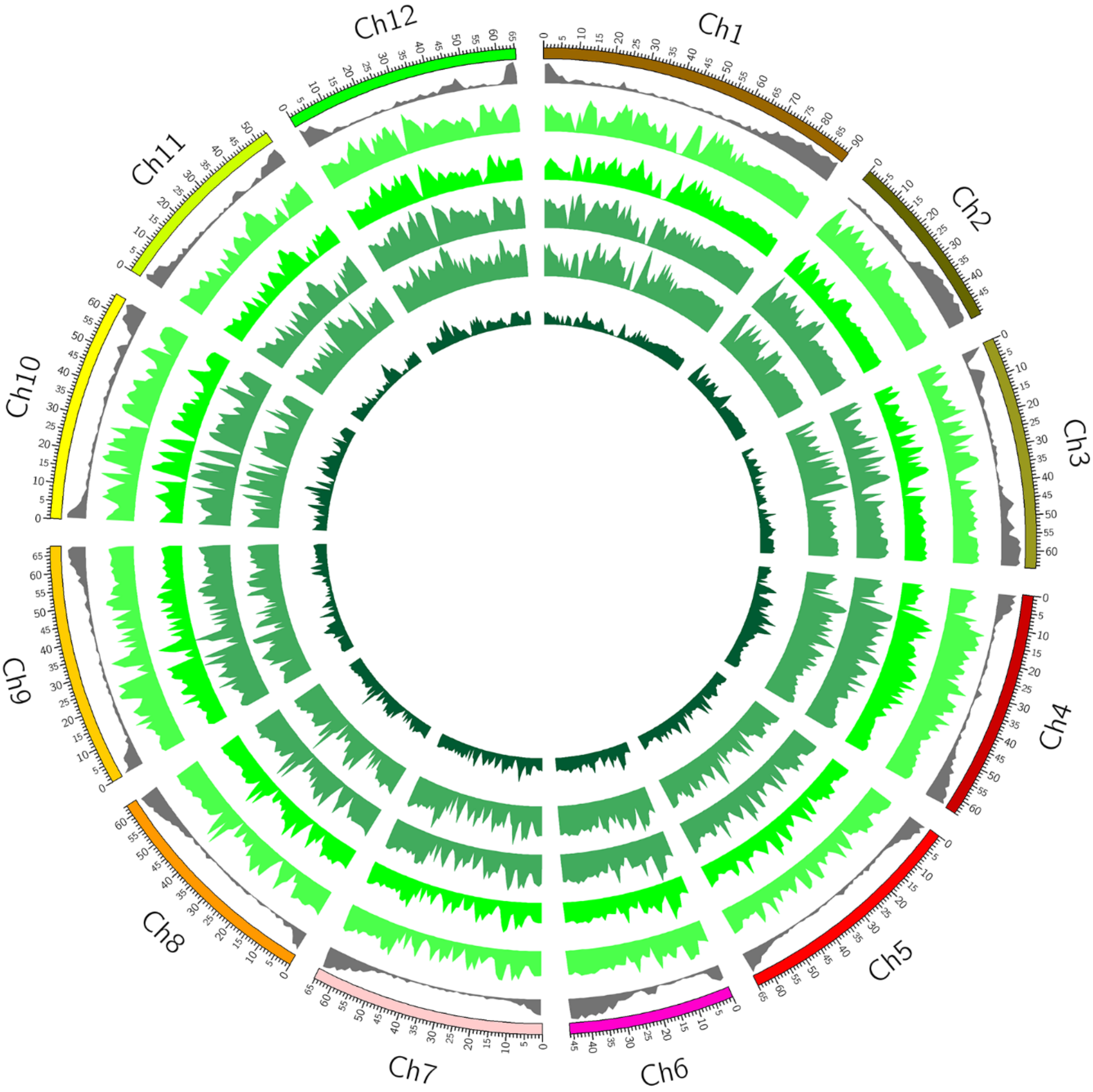

Fig. S1 - SNPs distribution of wild tomato on reference tomato chromosomes. The gene and SNPs density have been plotted in 1Mb sliding window using Circos. The tracks from outside to inside are; chromosomes of tomato; distribution of genes (gray) on reference tomato (genes per Mb max = 180); 5 circles of SNPs distribution (max = 16433); common SNPs of *S. chmielewskii*; common SNPs of *S. neorickii*; SNPs of *S. arcanum* (LA2172); SNPs of *S. arcanum* (LA2157); common SNPs of *S. arcanum*

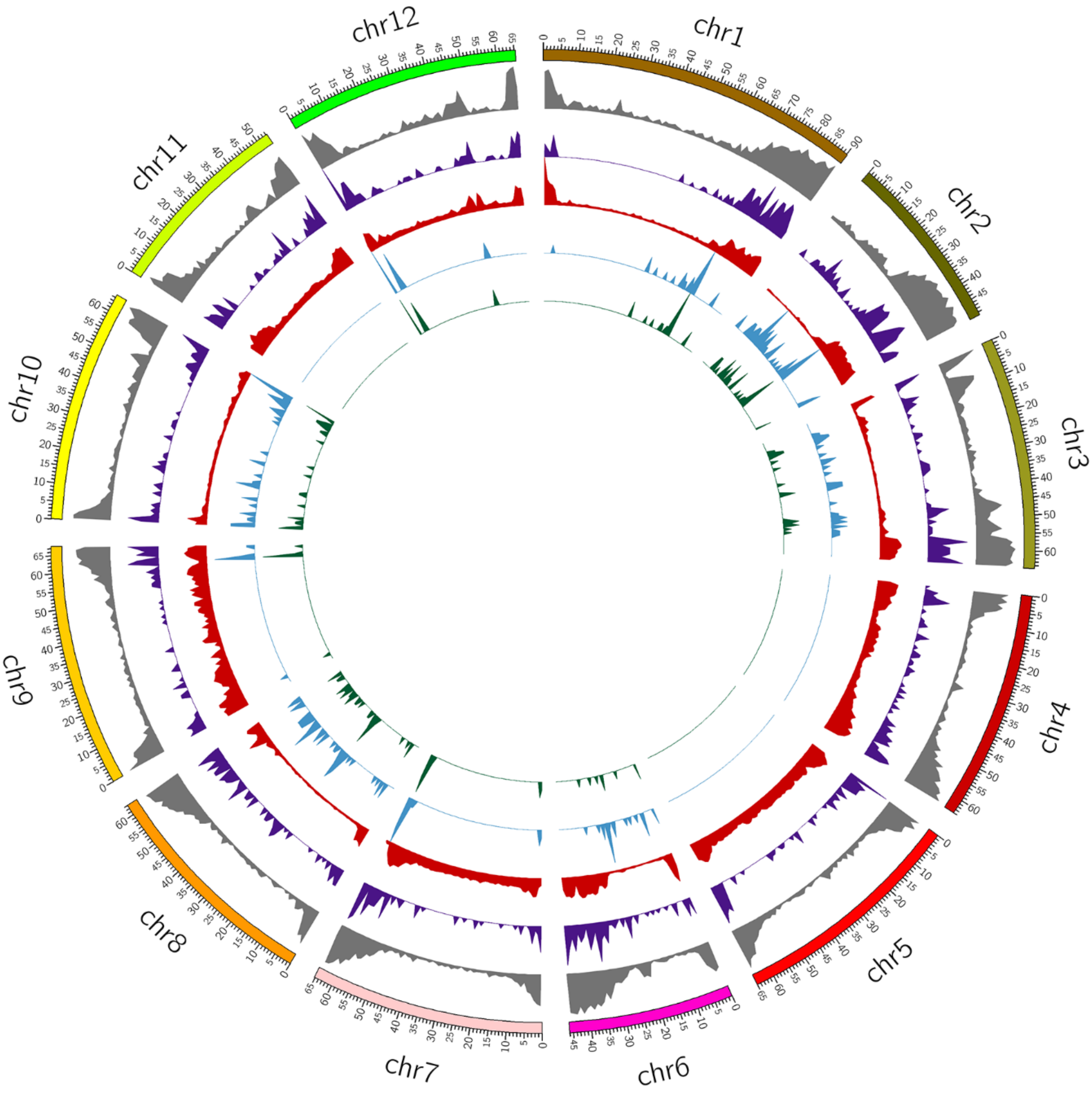

Fig. S2 - Distribution of common InDels of wild tomato species and InDels of cultivated tomato on reference tomato chromosomes. The gene and InDel density have been plotted in 1Mb sliding window using Circos. The tracks from outside to inside are; chromosomes of tomato; distribution of genes (gray) on reference tomato (genes per Mb max = 180); common InDels (purple) in 29 accessions of 12 wild tomato species (max = 11); unique InDels (red) present in all cultivated tomato accessions (max = 3867); common InDels (blue) present in 29 accessions of 12 wild tomato but not in present in 40 cultivated tomato accessions (max = 7); common genic InDels (dark green) present in 29 accessions of 12 wild tomato but not present in 40 cultivated tomato accessions (max = 6)

Table S1 - High quality variations of one accession each of 12 wild tomato species

Wild tomato species	Accession	Total variation	SNPs	InDels
S. pimpinellifolium	LA1578	2838469	2584661	253808
S. cheesmaniae	LA1401	3344138	3040651	303487
S. galapagense	LA1044	3528515	3186191	342324
S. chmielewskii	LA2663	6781227	6158734	622493
S. arcanum	LA2157	6444990	5860021	584969
S. neorickii	LA2133	6654142	6052131	602011
S. huaylasense	LA1983	5224527	4709066	515461
S. peruvianum	LA1954	5010193	4508697	501496
S. corneliomuelleri	LA0118	4543314	4085465	457849
S. chilense	CGN15530	4862282	4356811	505471
S. habrochaites	LYC4	4603233	4114640	488593
S. pennellii	LA0716	4678759	4198202	480557

Table S2 - Percentage of common variation within a species

Sn	Species	Accessions	SNPs	Common SNPs	Percent common SNPs from average SNPs of all accessions
1	***S. pimpinellifolium***	LA1578	2584661	2022949	
		LA1584	3422233		
		LYC2798	3744725		
		avg	3250540		62.23
2	***S. cheesmaniae***	LA1401	3040651	2061739	
		LA0483	3183211		
		avg	3111931		66.25
3	***S. galapagense***	LA1044	3186191	3186191	
4	***S. chmielewskii***	LA2663	6158734	5518570	
		LA2695	6005476		
		avg	6082105		90.73
5	***S. arcanum***	LA2172	5767870	2688214	
		LA2157	5860021		
		avg	5813946		46.24
6	***S. neorickii***	LA2133	6052131	4794303	
		LA0735	5806078		
		avg	5929105		80.86
7	***S. huaylasense***	LA1983	4709066	1317552	
		LA1365	3876168		
		LA1364	4036581		
		avg	4207272		32.64
8	***S. peruvianum***	LA1278	4306395	2286029	
		LA1954	4508697		
		avg	4407546		51.87
9	***S. corneliomuelleri***	LA0118	4085465	4085465	
10	***S. chilense***	CGN15532	3691138	2513385	
		CGN15530	4356811		
		avg	4023975		62.46
11	***S. habrochaites***	LA1718	3970472	1523960	
		PI134418	3934970		
		CGN157592	4168078		
		LA0407	4115389		
		LYC4	4114640		
		CGN157591	4243056		
		LA1777	3326696		
		avg	3981900		38.27
12	***S. pennellii***	LA1272	4186403	1190483	
		LA0716	4198202		
		avg	4192303		28.40

Table S3 - Common genic and genomic variations in each wild tomato

Sn.	Wild Tomato species	Genomic	Genic	%
1	*S. pimpinellifolium*	2164899	181859	8.40
2	*S. cheesmaniae*	2230113	199086	8.93
3	*S. galapagense*	3528515	320561	9.08
4	*S. chmielewskii*	6028997	1117637	18.54
5	*S. arcanum*	2921448	625680	21.42
6	*S. neorickii*	5205911	1001618	19.24
7	*S. huaylasense*	1434106	359972	25.10
8	*S. peruvianum*	2490685	563946	22.64
9	*S. corneliomuelleri*	4543314	873245	19.22
10	*S. chilense*	2750854	601388	21.86
11	*S. habrochaites*	1665632	717298	43.06
12	*S. pennellii*	1300315	438205	33.70

Table S4 - Common high quality SNPs within each species of wild tomato (presented as separate file in additional file 2)

Table S5 - Genes having species specific nucleotide variations in each of twelve wild tomato species (presented as separate file in additional file 2).

Table S6 - Number of SNPs and InDels per 1000 bases chromosome

Chromosome	No. of SNPs	No. of InDels	Chromosome size	SNPs/1000 bases	InDels/1000 bases
SL2.40ch01	229859	48480	90304244	2.55	0.54
SL2.40ch02	118069	29701	49918294	2.37	0.59
SL2.40ch03	164867	31952	64840714	2.54	0.49
SL2.40ch04	475192	61734	64064312	7.42	0.96
SL2.40ch05	638255	67190	65021438	9.82	1.03
SL2.40ch06	166536	34725	46041636	3.62	0.75
SL2.40ch07	567362	56214	65268621	8.69	0.86
SL2.40ch08	157888	24736	63032657	2.50	0.39
SL2.40ch09	839968	77300	67662091	12.41	1.14
SL2.40ch10	159808	22367	64834305	2.46	0.34
SL2.40ch11	327554	43352	53386025	6.14	0.81
SL2.40ch12	314258	38726	65486253	4.80	0.59

Table S7 - List of genes with common Variations (SNPs and InDels) present in all wild but not present in any cultivated tomato (presented as separate file in additional file 2)

Table S8 - List of genes with high Impact common wild variant alleles (presented as separate file in additional file 2)

Table S9 - Variations (present in all wild but absent in all cultivated tomato) and positions of variations in R genes with gene coordinates and gene annotation (presented as separate file in additional file 2)

Table S10 - List of genes with less then Tajima's D value -3 (presented as separate file in additional file 2)
